# Pathogenicity and virulence of human adenovirus F41: Possible links to severe hepatitis in children

**DOI:** 10.1080/21505594.2023.2242544

**Published:** 2023-08-06

**Authors:** Roger J. Grand

**Affiliations:** Institute for Cancer and Genomic Science, the Medical School, University of Birmingham, Birmingham, UK

**Keywords:** Adenovirus F41, adenovirus F40, HadV-F41, childhood hepatitis, hepatitis of unknown cause, adeno-associated virus 2

## Abstract

Over 100 human adenoviruses (HAdVs) have been isolated and allocated to seven species, A-G. Species F comprises two members-HAdV-F40 and HAdV-F41. As their primary site of infection is the gastrointestinal tract they have been termed, with species A, enteric adenoviruses. HAdV-F40 and HAdV-F41 are a common cause of gastroenteritis and diarrhoea in children. Partly because of difficulties in propagating the viruses in the laboratory, due to their restrictions on growth in many cell lines, our knowledge of the properties of individual viral proteins is limited. However, the structure of HAdV-F41 has recently been determined by cryo-electron microscopy. The overall structure is similar to those of HAdV-C5 and HAdV-D26 although with some differences. The sequence and arrangement of the hexon hypervariable region 1 (HVR1) and the arrangement of the C-terminal region of protein IX differ. Variations in the penton base and hexon HVR1 may play a role in facilitating infection of intestinal cells by HAdV-F41. A unique feature of HAdV-F40 and F41, among human adenoviruses, is the presence and expression of two fibre genes, giving long and short fibre proteins. This may also contribute to the tropism of these viruses. HAdV-F41 has been linked to a recent outbreak of severe acute hepatitis “of unknown origin” in young children. Further investigation has shown a very high prevalence of adeno-associated virus-2 in the liver and/or plasma of some cohorts of patients. These observations have proved controversial as HAdV-F41 had not been reported to infect the liver and AAV-2 has generally been considered harmless.

## Introduction

Human adenoviruses (HAdVs), together with adenoviruses infecting other mammals, are members of the genus *Mastadenovirus*. They were first isolated in 1953 from human adenoids, following an outbreak of respiratory disease in a military facility [1]. Since then, over 100 different human adenoviruses have been characterized. They have been divided into seven species or groups, A to G. Viruses within a species show a marked similarity in nucleotide sequence and in tropism for site of infection. Initial subtyping was on the basis of haemagglutination inhibition and serum neutralization assays, but more recently genetic analysis has been used to assign viruses to a particular species [2–4]. Most HAdVs have been allocated to species D which has 75 members at the last count; species A comprises 4 viruses, species B 16 viruses, species C 5 viruses, species F 2 viruses, and E and G, 1 member each. In the family *Adenoviridae*, as well as mammalian viruses (*Mastadenovirus*), there are also adenoviruses which infect birds (*Aviadenovirus*), and fish (*Ichtadenovirus*). In addition, two other genera have been characterized *Atadenovirus* contains a mixture of adenoviruses infecting birds, mammals, and reptiles and *Siadenovirus* contains viruses which infect birds and amphibians [5].

The HAdVs are non-enveloped DNA viruses with a linear double stranded genome of approximately 30–40 kbp [6,7]. They tend to be restricted in the tissues and organs they infect, and this limitation results in specific adenovirus-associated diseases [8–10]. Thus, species B, C, and E target the respiratory tract and cause pneumonia and acute respiratory infection; species C also causes hepatitis and pharyngitis; species B and D infect the eye and cause keratoconjunctivitis and species B has also been linked to renal and urinary tract infections; species A, F, and G target the gastrointestinal tract and cause gastroenteritis and diarrhoea. Of the viruses causing gastrointestinal disease, the group F adenoviruses are most common. Species F has two members, HAdV-F40 and HAdV-F41. Together, they are known as the enteric adenoviruses and are the second most common cause of viral gastroenteritis and diarrhoea in children, worldwide.

Recently, HAdV-F41 has also been linked to a large number of cases of acute, severe hepatitis “of unknown origin” in young children. The nomenclature “hepatitis of unknown origin,” which is used throughout this review, is taken to mean that the patients are negative for hepatitis viruses (HAV, HBV, HCV, HDV, and HEV) and have serum aspartate aminotransferase or alanine aminotransferase level over 500 IU/L. This outbreak is considered, in detail, towards the end of this review (Section 8).

The enteric adenoviruses were first detected in stool samples from children suffering from acute gastroenteritis [11,12] and were later shown to comprise two closely related subtypes, HAdV-F40 and HAdV-F41 [13,14]. Complete nucleotide sequences are now available for several isolates of each virus: for example, HAdV-F40 Duggan (L19443) and HAdV-F41 Tak (DQ315364). HAdV-F40 and HAdV-F41 comprise 34,214 and 34,188 nucleotides, respectively [15,16]. Although the species F viruses are of considerable clinical importance, they have not been subject to the same detailed virological and biochemical analysis as the much more commonly studied species C (for example, HAdV-C2 and HAdV-C5) or species A (for example, HAdV-A12) viruses. This is partly because they are more difficult to propagate in the laboratory. Early studies showed that HAdV-F40 and F41 would grow well in certain commonly used cell lines, such as HT-29, but very poorly in others, such as HeLas. This led to them being termed “Fastidious viruses”; this is discussed in more detail in Section 4 of this review. The possible link of HAdV-F41 to hepatitis has stimulated scientific interest in the species F viruses, such that a cryo-EM structure has now been determined for the virus [17,18]. However, our knowledge of the properties and mode of action of individual HAdV-F41 proteins is very limited and, in most cases, non-existent. Therefore, some of the following discussion is based on extrapolation from our much more extensive knowledge of other HAdV species, in particular HAdV-C5. Several early reviews of HAdV-F species viruses contain useful information [16,19–23].

## Species F adenovirus-associated disease

Infection with human adenoviruses is very common with most children infected with at least one species in early childhood. In immunocompetent individuals, symptoms are generally mild and disappear within one to two weeks. Simplistically, infection with adenoviruses from different groups is associated with particular clinical outcomes [9,10]. For example, respiratory disease, such as pneumonia, is mainly associated with species HAdV-B, C, and E, whereas keratoconjunctivitis is caused by species HAdV-B and D. Viral gastroenteritis and diarrhoea are predominantly caused species by HAdV-A, HAdV-F, and HAdV-G adenoviruses although it is notable that other adenovirus species can be shed in the gut (see Section 4).

The enteric, species F, adenoviruses cause gastroenteritis and diarrhoea in young children and infants throughout Europe, Africa, Asia, and the Americas, and are linked to 5–20% of the cases of diarrhoea [16]. They cause disease equally in industrialized and developing countries with little seasonal variation. An incubation time of about 1 week has been observed, after which infected children suffer from diarrhoea and vomiting but generally do not have fever. The two adenoviruses cause similar disease although HAdV-F40 infection has been suggested to result in milder diarrhoea symptoms [16]. In addition, both viruses tend to cause a milder disease than rotavirus, the primary cause of viral gastroenteritis in children. Transmission of HAdV-F viruses is probably by faecal-oral spread. Although most infected children recover quickly, enteric adenoviruses occasionally have been associated with fatal disease [24]. Infection with the HAdV-F species virus is common in children but is much rarer in adults. For example, in a wide-ranging study, in Brazil, of adenoviruses present in faecal samples from 5035 patients with gastroenteritis, the most common were enteric viruses (78% of the adenovirus-positive isolates, with 72% species F) [25]. The great majority of adenoviruses were present in the samples from children under 5 years of age (82%) with only 4.5% of the adenovirus-positive isolates from individuals over the age of 25 [25]. The mean and median ages of HAdV-positive patients in this study were 5.9 and 1 year, respectively.

In immunocompromised patients HAdV-F40 and 41 infections have not been commonly reported. For example, in a study of 289 immunocompromised patients only five were found to be infected with enteric adenoviruses [26]. In studies of AIDS patients, there was a weak correlation between increased shedding of enteric viruses and immunodeficiency although no specific virus was linked to AIDs-associated diarrhoea [27,28]. In a study of stem cell transplantation in children, adenovirus was isolated from 40% of the patients, but no species F viruses were observed [29]. Similarly, in a study of 153 transplant patients, adenovirus was isolated from the stool of 53 individuals. On genotyping the viruses, 76% were species C, 11% species A, 7% species D, 4% species B, and only 2% species F [30]. It has been concluded that in transplant patients there is “a very strong preponderance of species C in most instances” [31]. The corollary of this appears to be that immunosuppression makes very little difference to the incidence of HAdV-F40 or HAdV-F41 infection. In addition, transplantation tends not to occur in very young children who constitute most patients suffering from enteric adenovirus-associated gastroenteritis. It might be supposed that patients who are immunosuppressed will have passed the age when HAdV-F adenovirus infection is prevalent. A possible association of HAdV-F41 with severe, acute hepatitis in young children is considered in detail in Section 8.

## Viral infection and persistence

Although most cases of adenovirus-attributable disease arise from *de novo* infection, it is now clear that the virus can persist in individuals in a latent form (reviewed [31,32]). Several sites of viral persistence have been identified. It was originally shown that group C adenoviruses persist in adenoids and tonsils [33] but adenoviruses have also been reported in other sites, such as lung epithelial cells and, importantly, the intestine [34–37]. Most studies of adenovirus persistence have concentrated on the species C types, such as HAdV-C2 and HAdV-C5. Adenoviruses were originally isolated from tonsils and adenoids [1]. Slightly later studies showed that small amounts of the group C viruses, which were able to replicate, could persist in these tissues [38–41]. However, it was also noted that in many cases adenoviral DNA could be isolated from tonsils, but this was not associated with infectious virus, leading to the suggestion that mucosal-associated lymphoid tissues were sites of adenovirus latent infection [42]. In a further study using material obtained from 203 patients undergoing tonsillectomies, it was shown that only a small proportion of samples (less than 15%) contained replicating virus; however, stimulation *in vitro* led to viral replication in most cases [41]. By the age of four, it appears that almost all children are positive for adenovirus DNA in their lymphoid cells, declining to around half by the age of 16 [41].

There is also strong evidence that the gastrointestinal (GI) tract can serve as a site of adenovirus persistence, particularly in children [32,35,43]. Shedding of adenovirus in the stool of adenovirus-infected individuals has been observed months after signs of infection had disappeared. Significantly, based on data obtained from screening paediatric transplant recipients, it was seen that those developing viraemia had detectable adenovirus in their stool samples before it could be observed in peripheral blood [44,45]. In a more detailed study of children receiving allogeneic stem cell transplantation, it was observed that adenoviremia was almost always preceded by the detection of adenovirus in stool samples [30]. However, many patients had adenovirus present in their stool who did not develop invasive disease [30]. These and other studies strongly suggest that the intestine probably serves as the primary site for general adenovirus persistence or latency. It is likely, particularly in view of their main sites of infection, that this is the case for the HAdV-F species viruses. However, when the relative prevalence of HAdV species in the GI tract has been analysed group C viruses have been found to predominate [30,35]. Similarly, in the analysis of stool samples from transplant patients, group C viruses were again the most common (species A, 11%; B, 4%; C, 76%; D, 7%; and F, 2% [43]). Although the group C viruses have been most commonly identified in association with the gastrointestinal tract, it seems reasonable to suggest that this is the site of persistence of all types of HAdV, including the species F, particularly as this is considered to be the primary site of infection by HAdV-F40 and HAdV-F41.

In a further study comparing HAdV present in the GI tracts of paediatric patients undergoing haematopoietic stem cell transplantation (HSCT) with a similar number of non-HSCT patients, HAdV was detected throughout the intestine but was most common in the ileum of about one-third of immunocompetent patients tested. Analysis of the biopsy material showed its presence in mucosal lymphocytes but not in epithelial cells. However, when the analysis was carried out on patients undergoing transplants, very large accumulations of HAdV were seen in epithelial cells [35]. This led the authors to propose that human adenoviruses persist in intestinal lymphocytes over long periods but cannot efficiently replicate in them. However, small numbers of viruses, leaking from there, can enter and replicate in proximal epithelial cells - these viruses are then detectable in stool. In immunosuppressed individuals, replication in epithelial cells increases to a very high level [43]. To what extent any or all of these observations apply to the HAdV-F40 and HAdV-F41 is not clear at present, but as this is the primary site of infection it would be surprising if the enteric viruses did not persist there.

**Figure 1.**
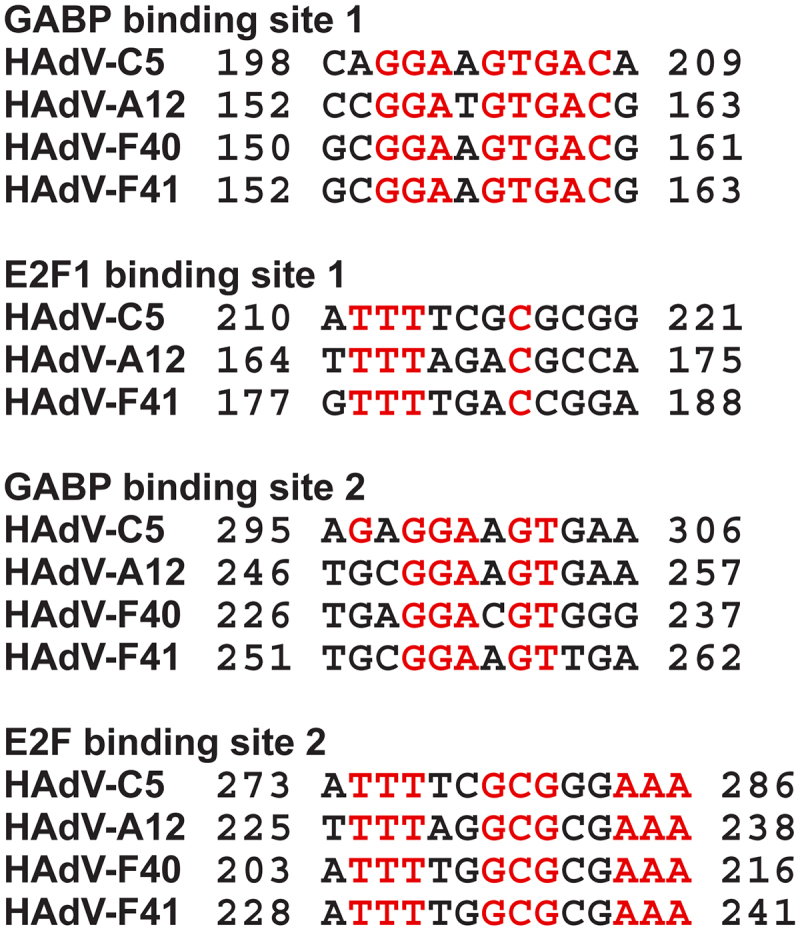
Nucleotide sequence alignment of the GABP1 and E2F binding sites in the E1A enhancer regions from adenovirus species. The binding sites as designated in [46] are shown, together with the corresponding nucleotide sequences from HAdV-F40 and F41. Conserved nucleotides are in red.

Although the idea of adenovirus latency and persistence has been current for several decades, the mechanism responsible has received much less attention. However, in an elegant study by Zheng and colleagues using fibroblasts and human bronchial epithelial cells, it was shown that viral replication was appreciably attenuated in primary cells by interferons (IFN) [46]. IFNs block the binding of GA binding protein α/β (GABP) to the E1A enhancer region during the early stages of infection [46]. Mutational analysis showed that repression of E1A expression by IFNs requires an E2F binding site in the E1A enhancer; IFNs increased the level of E2F-associated Rb and p107 at the E1A enhancer. Addition of E1A 12S protein was sufficient to rescue HAdV replication by dissociating the E2F-Rb family protein interaction [46]. Although the study was carried out primarily using HAdV-C5 it was shown that the GABP and E2F/DP binding sites were all conserved in E1As from species A, B, D, and E viruses. Furthermore, IFNs were able to inhibit viral replication in human fibroblasts [46]. Comparison of the nucleotide sequences of HAdV-F40 and F41 E1A genes with those of HAdV-C5 and HAdV-A12 shows a high degree of homology in the first GA binding protein (GABP) binding sites and in the second E2F binding sites delineated by Zheng and colleagues (Figure 1). The second GABP site is also well conserved between the four viruses (Figure 1). However, there is less similarity when considering the 3’ binding site for E2F/DP (Figure 1 [46]). Indeed, it is not possible to identify an E2F/DP binding site 1 for HAdV-F40. It has been concluded that the enteric adenoviruses are also susceptible to IFN through binding to upstream sites in the E1A genes.

In a later report, it was shown that the HAdV-C5 E3-19K protein activates the unfolded protein response (UPR) sensor IRE1α in the endoplasmic reticulum (ER) [47]. This is specific as E3-19K has no effect on protein kinase R-like ER kinase or activating transcription factor 6 which are also UPR sensors. Activation of the IRE1α nuclease by E3-19K initiates splicing of X-box binding protein (XBP1) mRNA [47]. The association of XBP1 with the E1A enhancer/promoter stimulates E1A transcription, expression of E3-19K and viral infection. It has been concluded that the five components (E1A, E3-19K, IRE1α, XBP1s, and the E1A enhancer/promoter) constitute a feedforward loop sustaining persistent infection in the presence of IFN and lytic infection [47]. Whether a similar mechanism is active during species F virus infection is not known, but it seems reasonable to assume that it is. One point of interest is that the IRE1β ortholog is primarily expressed in the digestive tract, whereas IRE1α is expressed in most cell types. It is not clear whether IRE1β could function in the feedforward loop.

## Viral entry

**Figure 2.**
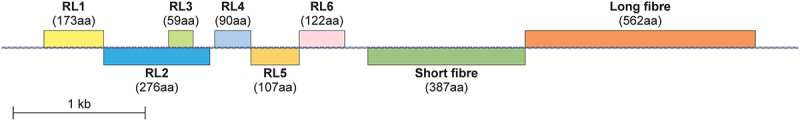
Locations of ORFs in the E4 genes of HAdV-F40. Locations of transcripts are shown as blocks. E4 transcripts are labelled with the number of constituent amino acids as in the original publication. The HAdV-F41 gene is arranged identically to that of HAdV-F40. Based on data from [16].

HAdV species A, C, D, E, and F gain entry to host cells by means of the widely expressed coxsackie-adenovirus receptor (CAR) [48,49]. This is recognised by the knob domain of the virus trimeric fibre capsid protein. The B group viruses are recognized by CD46 [50]; additionally, the glycans GD1a and polysialic acid, and desmoglein-2 can also act as receptors (reviewed [51]). CAR is an attachment receptor, but there is also a requirement for association with an entry receptor. For most adenoviruses, this is a member of the RGD-binding group of α5 integrins which bind to the viral penton base and act as secondary or accessory receptors, leading to internalisation of the virus. The group F viruses bind to the CAR receptor but, uniquely, they express two fibre proteins, encoded by adjacent genes (Figure 2). One is similar to the molecules present on the surface of group A, C, and D adenoviruses (termed long fibre) whereas the other (short fibre) is of lower molecular weight and does not bind CAR but probably interacts with heparan sulphate [53,20,54]. HAdV-F40 and HAdV-F41 make use of laminin-binding integrins, most probably α6β4, rather than the RGD-binding integrins [55]. The necessity for a different co-receptor is due to sequence variations in the penton base of these viruses. Thus, the RGD motif present in almost all human adenoviruses is replaced by an RGAD motif in HAdV-F40 and an IGDD motif in HAdV-F41 [55,56]. Interestingly, it was demonstrated that at low pH, as seen in the intestine, the interaction between the long fibre and CAR was inhibited, although the association of the short fibre with heparan sulphate was enhanced [54]. The possibility that the short fibre could act as the accessory receptor, rather than the penton base, has been largely discounted [49,55,57]. Thus, it has been suggested that, as small intestine epithelial cells do not express RGD binding α5 integrins, the enteric adenoviruses have evolved to make use of integrins which are expressed. Laminin-binding integrins are generally expressed on the basal surface of epithelial cells of the intestinal lining; when the cells migrate from the crypt they are shed from the villi, leaving gaps which may expose integrins and CAR molecules on the basal and lateral surfaces of the intestinal cells [55,58,59]. Other molecules on the cell surface have also been implicated in adenovirus attachment, such as desmoglein-2, sialic acid, heparan sulphate proteoglycans, GD1a glycans, MHC class I, CD80, and CD86, although it is not clear whether they are recognised by the enteric adenoviruses [60]. Although fibre and penton base are generally considered to be the major viral proteins involved in host cell recognition HAdV-C2/5 hexon interacts with the SR-A6 scavenger receptor [61,62:] [63]. However, this appears to occur in murine macrophages but not in human tumour cell lines such as A549.

Specific evidence relevant to the uncoating and trafficking of HAdV-F40 and F41, within the infected cell, is very limited; therefore, it has been assumed here that the mechanism involved in dissociation of HAdV-F41 is broadly similar to that described for other adenovirus species, generally HAdV-C2/5 (reviewed [64,65] [66]). Once the HAdV-C species virus has gained entry into the host cell by endocytosis, a series of changes take place in which the capsid essentially breaks down [64,65,67]. Binding of the penton base to the integrin initiates endocytosis and eventually lysis of the endosome [66]. Simplistically, at the plasma membrane protein IIIa, protein VIII, fibre and some of the protein V and penton base proteins are dissociated from the virion, over the course of about 15 min [66–68]. Acidification of the endosome leads to the dissociation of penton base and protein IX. Lysis of the endosome membrane, mediated by upstream actomyosin-drifting motions of virus particles bound to CAR, results in release of the remains of the viral particle into the cytoplasm. Protein VI separates and is released on break down of the endosome membrane, together with a proportion of protein V [69]. The transport of the remaining virion to the nuclear envelope or to the vicinity of the centrosome (depending on cell type) is facilitated by microtubule motors [70–73]; reviewed [74]. In non-polarised cells, dynein/dynactin-based transport leads to virion concentration near the centrosome. On the other hand, in polarised epithelial cells the kinesin system transports virions to the nucleus [75] reviewed [66,76]. Once at the nuclear membrane the virion binds to Nup214 through hexon [77–79]. At the NPC, the capsids are disassembled through the action of the cellular E3 ubiquitin ligase MIB1 [80,81]. Partial disruption of the capsid results from the action of kinesins. MIB1 then ubiquitylates protein V, leading to its separation from the capsid and the viral DNA. The resulting complex of viral DNA, HAdV terminal protein (TP), and protein VII is a nuclear import substrate recognised by the nuclear import machinery [66]. Although this general outline applies to the C species viruses, it is not clear if it is the mechanism adopted by the enteric viruses. Indeed, the B group viruses, such as HAdV-B7 and HAdV-B35, accumulate in lysosomes rather than being rapidly trafficked to the nucleus like HAdV-C5 [82]. Similarly, differences in the structure of the viral capsid proteins, such as protein IX, may have effects on the fate of the HAdV-F41 once it has entered the host cell [17,18]. Also, interaction with different integrins by the enteric adenoviruses could have consequences for the way in which the virion is trafficked in the infected cell. Answers to these questions will have to await further investigation.

## Species F adenoviruses as “fastidious viruses”

Historically, it has been reported that the enteric adenoviruses were restricted in their growth in human tumour cells, with notable differences between HAdV-F40 and F41 (reviewed [16,20,]). For example, in one report, appreciably lower titres of HAdV-F40, compared to HAdV-F41, were obtained after infection of WK conjunctiva cells, A549, HAdV-C5 E1HEK293 (293 cells) and KB cells [83]. Interestingly, HAdV-F40 did not replicate at all in HeLa cells in this study [83]. Other reports have indicated that neither virus can be grown in HeLa or KB cells, although this view is not universally held [13,20,83]. In other studies, it has been shown that both enteric viruses can be propagated in Chang conjunctival cells, 293 cells, and HT-29 and H1299 cells although generally less well than other adenovirus species (summarised in [16,20]). Again, conflicting reports have suggested that neither virus can be propagated in HT-29, PC, A431, or Tera-2 cells [16]. It is difficult to draw firm conclusions from these and many other studies although it is clear that the enteric viruses generally do not grow as well, under laboratory conditions, as adenoviruses from species A-E. One reasonably consistent result, however, is that HAdV-F41, and probably HAdV-F40, can be propagated in 293 cells. This is likely due to the helper function of the HAdV-C5 E1B55K protein, supplied in *trans* (reviewed [16]). In a detailed comparison of the infection of HeLa and 293 cells by the enteric viruses, it was observed that while most 293 cells could be infected, replication occurred in less than a fifth of the HeLa cells [84]. Furthermore, although the virus yield was similar for both cell lines, progeny viruses were only released from 293 cells. An extremely high particle-to-infectivity ratio was observed for the enteric viruses (100–1000-fold greater than HAdV-C5), although the yield of virus particles was similar for the three viruses [84]. It is likely that the very high ratio and the failure to release virus particles, seen with HeLa cells, may explain, at least partially, the difficulty in propagating the enteric viruses in the laboratory.

More recently, 293-based cell lines, which also express HAdV-C5 E4orf6, have been used to propagate HAdV-F viruses more successfully [85,86]. As both HAdV-C5 E1B55K and E4orf6 increase growth and propagation of fastidious adenoviruses, it is possible that the viruses lack, or have restricted, ability to recruit the cellular E3 ubiquitin ligases necessary for replication of almost all adenoviruses, although this has not been demonstrated [87]. Of course, both HAdV-F40 and F41 appear to have no difficulty in growing and replicating in the gastrointestinal tract of infected children. It might be assumed that a function supplied by the HAdV-C5 E1B55K and E4orf6 proteins is endogenously present, although there appears to be no evidence for that. Similarly, the apparent lack of an adenovirus death protein may reduce the release of progeny virus from infected cells in cell culture experiments (see section 6 and [52]). The rapid turnover of HAdV-F virus infected mucosal cells in the intestine might also facilitate the release of virus progeny without a highly developed mechanism for cell lysis.

## HAdV-F40 and HAdV-F41 genomes and the roles of the viral early proteins

A complete nucleotide sequence for an enteric adenovirus, HAdV-F40, was first published in 1993 [15,16]. Since then, sequence data has become available for numerous strains of HAdV-F40 and HAdV-F41 and these have been deposited in appropriate data bases (for example, HAdV-F40 strain “Dugan,” L19443 and HAdV-F41 strain “Tak,” DQ315364.2). The notable increase in interest in HAdV-F41, initiated by the suggestion that it could be responsible for the recent outbreak of acute childhood hepatitis (discussed in detail in Section 8), has led to the sequencing of multiple viral isolates (for example [88]). However, to date, no consistent mutations in the HAdV-F41 genome have been detected, which are specifically associated with the childhood hepatitis (Section 8). The genomes of the two enteric adenoviruses are similar to each other with about 80% homology. Apart from that, the HAdV-F41 genome is more similar to the species HAdV-A than to any other species, with the possible exception of HAdV-G52 (the single member of that species). Comparisons of the sequences of several adenovirus genes (for example, hexon, L1, L2, penton base, and E4orf6) have been carried out, and in each case similarities of HAdV-F40 and F41 to species A have been appreciably greater than for species B, C, and D orthologs [89–93].

Detailed analysis of 65 available complete HAdV-F41 genomes has allowed the separation of three lineages [88]. Lineage 1, comprising seven members (one of which was the original “Tak” strain) had 99.3% average nucleotide identity and derived from several geographic locations. Lineage 2, with 53 members, again originated from multiple countries and had 99.8% nucleotide identity. Lineage 3 also had very high nucleotide identity but diverged from lineages 1 and 2 (98.2% identity). The most notable differences between the lineages were seen as a 45-nucleotide deletion in the lineage 3 long fibre gene, together with 21 SNPs. Four amino acid substitutions were seen in lineage 3 fibre knob region [88]. There was also divergence in the lineage 3 short fibre gene, which resulted in 20 amino acid substitutions. Considerable variations between the lineages were also reported for the E3 and E4 regions. Differences between the lineage 3 E3 region and a reference HAdV-F41 genome were greater than between the “Dugan” HAdV-F40 and “Tak” HAdV-F41 strains. Lineage 1 had an appreciable number of SNPs in the E4 region compared to the reference sequence [88]. Unfortunately, only one sequence of HAdV-F41 from a child suffering from severe hepatitis was available for this study and that was most similar to lineage 2.

The overall organisation of the genomes of the species F adenoviruses is similar to species C and A viruses, with a few notable exceptions. The E1 regions of HAdV-F40 and F41 are 80% homologous and have 52% homology to HAdV-C5 [16]. HAdV-F40 and HAdV-F41 E1A proteins have similar conserved regions (CRs) to other adenovirus E1As but have only about 50% homology to the HAdV-C5 protein [94,95]. Interestingly, differences between HAdV-F41 and a large number of other E1As have been noted in very highly conserved residues in the second Rb binding site (which overlaps the N-terminal region of CR1) and in a highly conserved CBP/p300 binding motif at the C-terminal end of CR1 [94]. It is not clear what effect these differences, which are not conserved in HAdV-F40 E1A, makes to the virus and its ability to replicate. The E1B55K proteins from the two enteric viruses are highly homologous (90%) with almost all differences localized to the N-terminal fifth of the protein. Comparison with the sequences of E1B55K from other species shows a reasonable level of homology, with most differences, again, occurring in the N-terminal quarter [87]. Although the binding sites on E1B55K for cellular proteins have not been mapped in detail, it seems likely that the enteric virus E1B55K proteins associate with many of the same cellular targets identified for species A and C adenoviruses. Thus, HAdV-F40 E1B55K and E4orf6 can degrade MRE11, p53, and DNA Ligase IV [96]. Presumably similar activities can be attributed to HAdV-F41 proteins, although this has not been demonstrated. HAdV-F40 E1B55K can, like the protein from most other species, interact with p53 and inhibit its transcriptional activity [97]. Interestingly, HAdV-F40 and HAdV-F41 E1B55K can complement HAdV-C5 E1A in transformation assays almost as well as HAdV-C5 E1B55K [16,97]. However, the enteric virus E1A genes are unable to co-operate with E1B genes from HAdV-C5, HAdV-A12, or HAdV-F41 to transform BRKs fully, although they can co-operate effectively with activated *ras* [98]. It is possible that this is due to the differences in the E1A binding sites, reducing the ability of E1A to interact with and inactivate Rb family proteins and CBP/p300, although this has not been confirmed.

**Figure 3.**
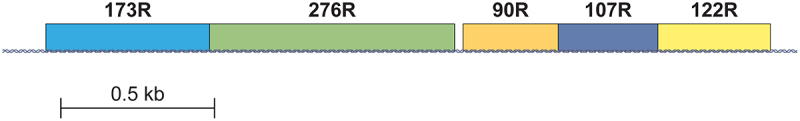
Locations of ORFs in the E3 and fibre genes of HAdV-F41. Locations of transcripts are shown as blocks. E3 transcripts are labelled RL1–6. The constituent amino acid numbers are shown. Based on data from [52].

The E2 regions encode the viral DNA binding protein (DBP/E2A), the pre-terminal protein (pTP), and the viral polymerase (pol), and these are thought to be highly conserved in HAdV-F40 and F41. It is assumed that they function in the same way as their HAdV-C5 and HAdV-A12 counterparts. The E3 regions of the two enteric viruses are very similar but have differences to HAdV-C5 E3 (Figure 3) [16,52]. HAdV-F41 E3 encodes six open reading frames, RL1–6 [52]. RL1, RL2, and RL3 appear to have no equivalent in the species C adenoviruses and encode proteins of 173, 276, and 59 amino acids, respectively. RL3 is read in a different reading frame to the others. The three other ORFs are similar to the well-characterized HAdV-C2 proteins; thus, RL4 is homologous to HAdV-C2 10.4-kDa, RL5 is homologous to HAdV-C2 14.5-kDa, and RL6 is considered to be analogous to HAdV-C2 14.7-kDa protein (Figure 3). Comparable proteins are also expressed by species B and E adenoviruses [52]. Although RL1 is equivalent in location on the viral genome to HAdV-C2 gp19 it shares no obvious homology; however, it is approximately 30% homologous to the HAdV-A12 E3 264 residue protein. Similarly, RL2 appears to have no equivalent in HAdV-C2, HAdV-C5, HAdV-B3, or HAdV-E4 although it has approximately 34% homology to HAdV-A12 268 amino acid protein [16,52,99]. Based on a structural prediction, it has been suggested that HAdV-F41 RL3 is equivalent to HAdV-C2 E3 6.7-kDa protein although it has no sequence homology [52]. In HAdV-C5 proteins encoded by E3 protect adenovirus infected cells from killing mediated by cytotoxic T cells and toxic cytokines (see, for example, review by [100] [In the latter review a more modern nomenclature for the E3 proteins has been used, but in the present article we have retained the nomenclatures used in the original publications]). E3gp19K blocks MHC class I-restricted antigen presentation. The receptor internalization and degradation complex (RID) comprises E3–10.4kDa and E3–14.4kDa and controls degradation of surface receptors, such as Fas. E3 6.7-kDa acts with RID and may help to inhibit apoptosis. E3–14.7kDa is an also an inhibitor of apoptosis [100]. Adenovirus death protein (ADP) is encoded by the E3 region in HAdV-C2 and C5 viruses but appears to be absent from HAdV-F virus E3 regions. Similarly, no evidence of a “death like function” was found for HAdV-B1 virus [101]. Interestingly, the incorporation of the HAdV-C5 ADP gene into recombinant HAdV-F41 increased virus yield substantially through enhancing the spread of the progeny virus among packaging cells [102]. It has been suggested that the similarity of the enteric virus E3 regions to that of HAdV-A12 rather than the group C viruses, such as HAdV-C2 or 5, may be linked to a shared site of infection, the gastrointestinal rather than the respiratory tract [16].

The E4 regions of HAdV-F40 and HAdV-F41 are generally similar to those of HAdV-C2 and HAdV-A12 except that there is no equivalent of E4orf1 (Figure 2) [16]. However, it has been shown that HAdV-F40 can complement the HAdV-C2 deletion mutant *dl*808, which lacks most of the E4 region [103]. A major role of the adenovirus E4orf6 protein is, together with the viral E1B55K protein, to recruit a cellular ubiquitin E3 ligase, to ubiquitylate host proteins and, in many cases, target them for degradation by the proteasome [104] reviewed [87; 105]. HAdV-F40 E4orf6 forms a complex with the complementary E1B55K protein although some differences have been noted in their mode of action. The HAdV-F40 E4orf6 forms complexes containing Elongins B and C and E1B55K as is the case with HAdV-C5 and this is able to cause degradation of p53 and Mre11, as is the case for species A and C but not B, D, and E, adenovirus protein complexes [96]. A central component of the HAdV-C5 E1B55K/E4orf6 E3 ligase is cullin 5 (Cul5) [104]. However, it appears that HAdV-F40, and presumably HAdV-F41, recruit cullin 2, rather than Cul5, as is the case for species A adenoviruses [96,106]. The interaction of HAdV-F40 E4orf6 with Cul2 is due to the presence of a sequence resembling the cellular consensus Cul2 box; a similar sequence is present in HAdV-A12 E4orf6 [107]. As cullin 2 is part of the E3 ubiquitin ligase recruited by species A, F, and G adenoviruses, it has been suggested that this may be linked to their tropism for the gastrointestinal tract; thus, other human adenoviruses which target the respiratory tract or the eye do not have a Cul2 box and almost entirely recruit Cul5 [90]. Interestingly, in an extensive study, it was shown that all adenoviruses isolated from great apes encode E4orf6 proteins which bind Cul5, whereas those E4orf6 proteins isolated from monkey adenoviruses have a Cul2 box, suggesting that the ability to bind Cul5 coincided with the evolution of apes and hominids from monkeys [90]. It is not clear what advantage the use of Cul2 over Cul5 gives to the viruses. It has been shown, however, that cullin 2 is far more abundant in the intestine of the mouse than in lung tissue, whereas cullin 5 is present in both to an equal extent [90]. In humans, cullin 2 seems to be expressed at a reasonably high level in both respiratory and gastrointestinal tracts (https://www.proteinatlas.org/ENSG00000108094-CUL2/tissue) although cullin 5 appears to be particularly abundant in respiratory tract tissues (https://www.proteinatlas.org/ENSG00000166266-CUL5/tissue).

## The structure of adenovirus F41 and the roles of the viral late proteins

The overall structures have been determined for three human adenovirus types: HAdV-C5, HAdV-D26, and HAdV-F41; structures have also been determined for bovine BAdV-3, snake SnAdV-1, and lizard LAdV-2 [17,18,108–114]. All three HAdVs are broadly similar, with large, non-enveloped capsids of approximately 950 Å in diameter pseudo-T = 25 icosahedral particle with 12 trimeric hexons per facet (giving a total of 240 trimers) and pentameric pentons at the vertices. The hexons are stabilized by the minor capsid proteins-IIIa, and VIII. Proteins IIIa, VIII, fragments of VI and core protein VII are present in the interior of the capsid. Penton base subunits are organized into 12 homopentamers at the vertices of the capsid. These associate with the N-terminal regions of the trimeric fibre proteins. The N-terminal region of protein IX is present on the outer surface of the capsid between hexon trimers. The structure of HAdV-F41 has been solved, using cryo-electron microscopy to a resolution of 3.8 Å or 4 Å [17,18]. This was determined at both pH7.4 and pH4.0 [18], the latter to take into account the fact that the virus normally infects the GI tract; however, there appears to be little difference in the structure of the capsid at the lower pH [18]. On examination of the surface charge of the three human viruses at pH7.4 HAdV-D26 has been found to have a highly negatively charged surface, whereas HAdV-C5 only has negatively charged areas at the tops of the hexons; HAdV-F41 capsid is generally uncharged at pH7.4 and has two uncharged surface regions at pH 4.0. It was concluded that the icosahedral part of the capsid is unaffected by low pH, the virus having evolved so there are fewer charged amino acids on its surface compared to species C and D viruses, presumably as an adaptation to the acidic GI tract [17,18].

The major late genes present in the enteric viruses are comparable to those in other virus species. Hexon is 88% identical between the two viruses, which is similar to that seen within other species, such as HAdV-C. HAdV-F hexon most closely resembles that present in species A viruses. The major sequence difference between HAdV-F40 and HAdV-F41 hexons and HAdV-C5 hexon is in the loop regions exposed on the virus surface [115,116]. The overall structural fold of HAdV-F41 hexon is similar to that seen with other adenovirus species, although differences were observed in the seven hypervariable regions (HVRs), when comparing it to HAdV-C5 and HAdV-D26 [18]. In particular, HVR1 is much shorter and less charged in HAdV-F41 giving it a rigid structure, compared to HAdV-C5 [18]. Again, this may be an adaptation to the low pH found in the intestinal tract.

The L4 regions of HAdV-F40 and F41 resemble those seen for most other adenoviruses with the major similarity observed in the L4-100K protein. L4-33K and −22K are also suggested to be expressed by the enteric viruses [16]. As noted in Section 3 the penton base is involved in virus entry and binding to the accessory receptor. The penton base forms multiple interactions in the virion binding to hexon, fibre and IIIa protein [18]. Comparison of structures for the penton base determined in free solution and in the virion has indicated that the presence of other viral proteins causes four disordered regions to adopt defined conformations [18]. For example, the conserved random coil region between amino acids T^33^ and G^49^ is stabilized in the virion through association with two loop regions from protein IIIa [18]. Other regions of HAdV-F41 penton base which become structured in the virion are located between Y^419^ and L^429^ which becomes largely α-helical possibly through interactions in the capsid or binding to fibre. The two loop regions within the penton base V^70^ to N^110^ become structured through interaction with hexon molecules in the HAdV-F41 virion [18]. As mentioned in Section 3 the integrin binding RGD motif, present on the penton base of most adenoviruses, is not conserved in the enteric viruses but is replaced by IGDD in HAdV-F41 and RGAD in HAdV-F40. The loops containing the RGD sequence are disordered in HAdV-C5 and HAdV-D26 as is the IGDD sequence in HAdV-F41, presumably giving it some flexibility to allow binding to the host cell surface [17,18,109,114,117]. Overall, the structure of HAdV-F41 penton base is similar to that of the protein from species C and D [17,18]. However, it has been noted that the binding of penton to fibre may be different in HAdV-F41 and HAdV-C5 with less hydrogen-bridging and more hydrophobic interactions in the latter virus [17].

Two fibre proteins are expressed by the enteric adenoviruses-a “long fibre,” which binds to the CAR receptor and is similar to that present in all other human adenoviruses, and a “short” fibre which does not bind to CAR but to heparan sulphate [53,118,54,119]. This short fibre is considered to help protect the virus from the low pH found in the intestine. The major difference between the short and long fibres lies in the length of the shaft region between the tail and knob regions. In HAdV-F41 long fibre there are twenty-two 16 amino acid repeats, whereas there are only twelve repeats in the short form [119]. In the structural studies of the short fibre “head region” it was shown that there are distinct differences to the head regions of long fibres occurring in both HAdV-F41 and other adenovirus species, possibly explaining why there is no binding to CAR [120]. Whilst they are superficially similar, there is a specific deletion in the AB loop in the short fibre head, resulting in an appreciable conformational change, incompatible with interaction with CAR [120]. Other structural differences between the heads of the short and long fibres have also been reported [120].

A notable difference between HAdV-F41 and HAdV-C5 and HAdV-D26 is the arrangement of minor capsid protein IX on the surface of the capsid. In the case of the latter two viruses, it forms a highly ordered, tight triskelion-shaped network across the virion surface, locating in the valleys between the hexons; it also interacts and binds the hexons together imparting thermostability to the virus [109,110]; [17; 18]. In HAdV-F41, however, the structure of protein IX on the capsid surface is quite distinct and not so ordered [17,18]. It is probable that changes in the amino acid sequence of the protein are responsible for reorganization of protein IX. A model has been suggested in which HAdV-F41 protein IX forms four triskelions and three mobile helix bundles per facet [17,121]. In HAdV-C5 and D26, the helix bundles are not mobile [17]. Consistent with these data, in another investigation, it was observed that the C-terminal half of protein IX is flexible in HAdV-F41, exposing its C-terminus to the capsid exterior, unlike HAdV-C5 and HAdV-D26 [18].

Information for the minor capsid proteins IIIa, V, VI, VII, and VIII indicates that their structure and distribution in the virus particle is comparable for HAdV-F41 and HAdV-C5 [17,18]. Proteins IIIa and VIII line the internal capsid surface in HAdV-F41. Some VIII proteins interact with IIIa, stabilizing the vertices, while others maintain nonperipentonal hexons in the central plate of the facet [17]. In the cryo-EM study of HAdV-F41 it has been shown that the core protein V probably links the viral genome to the capsid; similar electron density was observed in the study of HAdV-C5 and HAdV-D26 [18,114,122].

Differences in the amino acid sequences and consequent structure of components of the adenovirus capsid determine differences in the abilities of various species to resist neutralization by human α-defensins (HDs). In most cases binding of defensin to the infecting adenovirus particle stabilizes the capsid and inhibits release of protein VI, blocking uncoating and exposure of the viral genome [123–126]. However, this is not the case for species D, and F viruses, which are resistant to HD5 and neutrophil peptide 1 (NP1) [126]. The ability of defensins to bind to the viral capsid is determined by a short four amino acid sequence in the N-terminal region of the fibre protein which is acidic in species A, B, and C HAdVs but positively charged and hydrophobic in HAdV-D and F viruses. The defensin also binds the penton base in the susceptible species, associating it with a disordered region at the top of the base. It was suggested that stabilization of the HAdV vertex region comprising the penton base and fibre can be achieved by a three-fold increase in intermolecular nonbonded interactions [126,127].

## Adenovirus F41 as a vector

Thousands of gene therapy trials have been or are being carried out with human adenovirus vectors (see for example [128] [129,130]). Most are for the treatment of cancer (oncolytic viruses). Others are for the use of adenovirus vectors as vaccines in which the vector expresses an antigenic protein or for gene therapy in which the vector expresses a protein to correct a genetic defect. It might be assumed that appreciable use would have been made of the species F adenoviruses to target intestinal cells, based on their tropism. This does not appear to be the case as most studies have used vectors based on HAdV-C5 (reviewed [131]). However, it has been shown that an HAdV-F41 vector binds to differentiated and undifferentiated enterocytes much better than a HAdV-C5 vector. In addition, the uptake of HAdV-F41 was much more efficient [132]. Unfortunately, these experiments do not appear to have been followed up in any systematic way.

## Adenovirus F41 and acute, severe childhood hepatitis

During the first six months of 2022 there were many reports of acute, severe hepatitis in young (under the age of five), otherwise healthy, children (reviewed, for example [133;] [134,135;] [136] [137; 138]; and references therein). Although most patients appeared to be in the U.K. and U.S., cases were seen in at least 30 countries, including Canada, Indonesia, Spain, Israel, Denmark, Ireland, France, Romania, and Belgium. The children presented with vomiting, jaundice, and diarrhoea. Overall, at least 1000 hospitalized children have been reported worldwide, some requiring liver transplants and a few dying. Although there was no obvious cause for the hepatitis that is not particularly unusual, as a direct cause for many cases of acute liver failure is often not found (see, for example [139,140]). However, for this cohort of children with acute hepatitis, almost all tested negative for hepatitis viruses (HAV, HBV, HCV, often HDV, HEV), Epstein–Barr virus (EBV), Cytomegalovirus (CMV), and Human Immunodeficiency Virus (HIV). Most patients had not been infected with SARS-CoV-2, nor had they been vaccinated against COVID-19. However, many of the children were positive for adenovirus. For example, in the UK Health Security Agency technical report published at the end of July 2022, summarizing cases in the U.K. up to that time, there had been 270 confirmed cases of severe hepatitis in children under the age of 10. Of these, 15 received liver transplants but none died. Almost all were tested for adenovirus and 65.9% were positive. Similarly, in a National Center for Immunization and Respiratory Diseases (NCIRD) report published in August 2022 (https://www.cdc.gov/ncird/investigation/hepatitis-unknown-cause/technical-report.html), summarizing the then current state of knowledge in 43 states of the U.S., it was reported that there were 358 patients under investigation with a median age of 2 years. At that time 6% had required liver transplants and 4% had died; 299 patients were tested for adenovirus and 45% were positive. A more detailed analysis showed that HAdV-F41 was, by far, the most common adenovirus. In one study from the U.K. 44 children (median age 4 years) with acute severe hepatitis were treated, of whom 6 required liver transplants. Of 29 patients who were tested, 93% were positive for adenovirus [141]. In another study of 15 children with acute severe hepatitis (median age 2 years 11 months), 9 had no obvious cause. Of these, eight tested positive for adenovirus, of whom five were positive for HAdV-F41 [142]. Thus, by late summer of 2022, HAdV-F41 was thought to be a likely cause of the hepatitis, even though it had not been detected in all the patients nor was there evidence of adenovirus-mediated tissue damage in many cases.

As the enteric adenoviruses show a marked tropism for the gastrointestinal tract and not the liver, this led to considerable confusion and debate. A further complication was added, towards the end of 2022 and in 2023, when three carefully performed, specific studies of young children, presenting with severe hepatitis with no known cause, showed the presence of adeno-associated virus-2 (AAV-2) in almost all members of the cohorts examined [143–145]. HAdV-F41 was detected in an appreciable proportion of patients but was not ubiquitous unlike AAV-2. Adeno-associated viruses (AAVs) require a “helper virus” to replicate; in these reports, it was suggested that the helper function was provided in some patients by adenovirus (generally HAdV-F41) but in others by Human herpesvirus 6B (HHV-6B). The results of these later studies are summarized in Table 1.

**Table 1.** AAV-2 and HAdV detected in cases of hepatitis of unknown origin. Summary of data from studies by [143; 145] and [144].

	Ho et al. 2023[143]	Servellita et al. 2023[145]	Morfopoulou et al. 2023[144]
Number of cases	32	16	38
Median age	4.1 years	3 years	<10 years
Number of controls	25 immunocompetent Including 12 HAdV positive controls	113	66 immunocompetent 21 immunocompromised
AAV-2 detected	9/9	13/14	27/28
HAdV detected	6/9	14/14 (10/14 HAdV-F41).	22/23
HHV-6B detected	3/9	7/14 (11/14 positive for EBV)	16/23
Viruses detected in the livers of patients	AAV-2 4/4	AAV-2 0/8 HAdV 3/8	AAV-2 5/5 HHV-6B 5/5
AAV-2 in controls	0/25	4/113	6/65 in blood of immunocompetent cases. 6/17 of immunocompromised cases.
HAdV in controls	0/13 in healthy controls 6/12 in HAdV-positive controls	9/113	1/65
HHV-6B in controls	0/13 in healthy controls 9/12 in HAdV positive controls.	1/113	0/2 livers
COVID-19 vaccination	0	Not known	Not known
SARS-Cov-2 infection	3/31 but 12/23 had evidence of past infection.	Not detected in cases or controls	15/20 seropositive
DRB1 × 04:01 allele	25/27 compared with 10/64 in controls	Not determined	12/13

Adeno-associated viruses are members of the *Parvovirus* family, of the genus *Dependovirus* [146–149]. They are small non-enveloped viruses, with a single stranded DNA genome comprising about 4.6K nucleotides. Approximately 100 genomic isolates and 13 serotypes have been identified. AAV is widespread in humans, with AAV-2 the most commonly identified and the most studied [150]. More than 90% of the adult population is naturally infected with AAV, with antibodies to the different AAV serotypes detected in most individuals [151–153]. Antibodies against AAV-2, for example, are present in 50–80% of the individuals although only about a third tend to be neutralizing antibodies [151,154]. The incidence of AAV-2 increases to approximately 90% in immunosuppressed individuals. Antibodies against AAV can be detected at birth, suggesting maternal transmission. Over the first year of life, antibody levels tend to fall and then rise until late adolescence [155]. It seems likely that AAV infection occurs again later in life, giving rise to increased seropositivity after the age of about 30 [156]. In studies of AAV distribution in human tissues, AAV *cap* gene sequences have been detected in brain, colon, liver, lung, kidney, spleen, and bone marrow, with the highest level in the liver and bone marrow [157]. Other reports have indicated the presence of AAV-2 in cervix, penis, uterus, abortion material, and blood [158–161]. High levels of AAV (predominantly AAV-2) are present in blood from about a third of healthy donors, with AAV residing in CD3^+^ T lymphocytes. This was thought to be a possible site for AAV persistence [162].

The consensus is that AAV is not associated with any disease and does not produce any clinical symptoms in the great majority of cases, although there is evidence for hepatitis following AAV gene therapy [163–169]. However, there have been suggestions of a link between AAV and cervical carcinoma, reproductive system disorders, and hepatocellular carcinoma (reviewed [170]). While some studies have indicated that AAV can have a protective role in cervical tumorigenesis, others have concluded that there is neither a positive nor negative link. Similarly, limited evidence has shown that AAV infection may contribute to placental complications and spontaneous abortions, whereas others have found no statistically significant difference between levels of AAV in spontaneous and therapeutic abortions [159,170]. There have also been suggestions that AAV could contribute to hepatocellular carcinoma by integration into the genomes of patients [171–174]. Insertion of AAV DNA was mainly in the *CCNA2, CCNE1, TERT, TNFSF10,* and *KMT2B* cancer driver genes, generally leading to protein over-expression. The original study by Nault and colleagues was the subject of strong criticism at the time, but a later widespread screen produced results consistent with the earlier investigation [171,172,175,176]. However, in studies of liver carcinomas from Korean, Thai, and Mongolian patients, the integration of AAV was at a very low level, leading the authors to conclude that AAV posed a minimal risk of causing hepatocarcinogenesis [177,178]. In immunocompromised individuals, levels of AAV tend to be higher (see [162] for example) but the virus still does not produce clinical symptoms.

Adeno-associated viruses require the presence of a co-infecting “helper” virus which is usually adenovirus or a herpesvirus although other viruses, such as HPV, can fulfil this role. Presumably to overcome the inefficiency of the requirement for infection of a host cell by two viruses at the same time, AAVs can establish a latent infection, which may, but often does not, involve integration of its genome into that of the host. The integration site has been mapped to chromosome 19q13.4, a site termed AAVS1 [179,180]. Commonly, the AAV-2 genome is present in human tissue samples, in the absence of a helper virus, as an extrachromosomal rolling circle. Activation of the virus requires expression of the viral Rep proteins, which are needed for replication, transcription, integration, and encapsulation. The *cap* gene encodes the three VP1, VP2, and VP3 proteins, which constitute the viral capsid and the membrane-associated accessory protein (MAAP) which aids AAV replication and has a role in controlling HAdV infection. The assembly of the capsid is facilitated by the assembly activating protein (AAP) [148,181–183]. Many host cell surface molecules are recognised by infecting AAV-2 (reviewed [184]). Glycans, which were once thought to be primary receptors, may now be considered low specificity attachment factors. Proteins constitute the most likely candidates for AAV receptors with knock-out of more than 50 genes affecting AAV resistance [184]. Adeno-associated virus receptor (AAVR, also known as KIAA0319L) is a widely expressed membrane protein, which is important for trafficking and likely serves as a viral entry receptor for AAV-2. The capsid spikes surrounding the threefold axes bind to the PKD2 motif in the ectodomain of AAVR [185–187].

Whilst the popular press and social media largely considered the mystery solved, with AAV-2 the cause of the hepatitis, several important unanswered questions remain. Firstly, although AAV-2 can infect liver cells, it is normally considered to be harmless and there are virtually no clinical conditions associated with “normal” AAV infection. Why should AAV-2 suddenly cause severe liver disease? Secondly, HAdV-F41 is well-known to infect the gut but not the liver; as discussed throughout this review, it is widely associated with gastroenteritis but not associated with hepatitis, even in immunocompromised patients. On a more general level, it is interesting to note that there have been virtually no reports of HAdV-F40 in the patients with hepatitis, which is remarkable as it is very similar to HAdV-F41 and is equally prevalent in the gut.

In an MMWR report, published 14 June 2022, based on results from four data sources in the U.S., it was concluded that there had been no significant increase in hospital admissions of children suffering from hepatitis over the period from January 2017 to March 2022. Nor had there been an increase in the frequency of liver transplants [188]. However, it was noted that there was a marked decrease in HAdV-F41 in stool specimens from children over the period May 2020 to August 2021, presumably corresponding to COVID-19 lockdown. From October 2021 the number of children positive for HAdV-F41 returned to the pre-pandemic level, slightly preceding the reports of the hepatitis [188]. This could imply that there has always been a cohort of children with severe hepatitis, of unknown origin, who were infected with HAdV-F41 and AAV-2 but went unobserved. However, in a large retrospective study of non-A-E-hepatitis in children covering the period from 1990 to 2022, very few cases of adenovirus infection were seen, although there was a marked increase in hepatitis cases from 2019 [189]. It was suggested that there is, indeed, an increased incidence of acute, severe hepatitis of unknown origin in children but several factors could trigger it, one of which could be HAdV-F41 and/or AAV-2 [189]. For example, an increase in HAdV-F41 and AAV-2 occurrences has been observed in wastewater in Northern Ireland, coincident with an increase in severe hepatitis in young children [190,191]. Although there was an increase in HAdV-F41 at the same time as the increase in hepatitis, the levels of other adenoviruses in the wastewater stayed approximately constant [190].

It is possible that a lack of exposure to micro-organisms in general and viruses in particular, such as HAdV-F41 and AAV-2, during COVID-19 lock-down could have meant that young children developed “an immunity gap” which has been defined as “a group of susceptible individuals who avoided infection and therefore lack pathogen-specific immunity to protect against future infection” [192]. Obviously, this could have contributed to the hepatitis. It is notable that an increase in childhood infection, generally, has occurred after the end of COVID-19 restrictions (for example [192,193] [194–196]). This has been most pronounced among young children.

In one of the latest studies, showing the presence of AAV-2 in the patients with hepatitis, it was noted that the patients had a high frequency of the MHC alleles HLA-DRB *04:01 (25/27 cases compared to 10/64 in controls), DQA1 × 03:03 (23/27 compared to 11/64 controls), and DRB × 01:03 (23/27 compared to 21/64 controls) [143]. In another study HLA-DRB *04:01 was also seen in 12/13 cases [144]. HLA-DRB *04:01 is known to be associated with autoimmune disease, and it was suggested that its presence could increase susceptibility to viral infection [143]. Further studies are required to examine links between viral infection, MHC alleles, and hepatitis.

**Figure 4.**
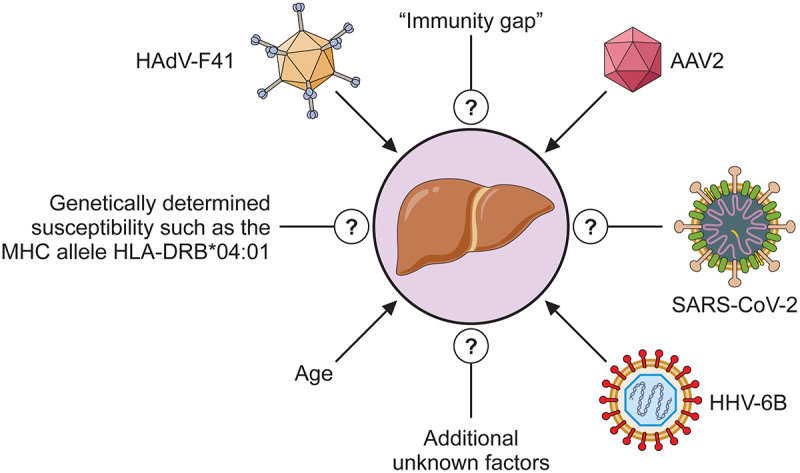
Cartoon showing possible factors contributing to severe acute childhood hepatitis “of unknown origin.”

Whether the hepatitis is caused by direct viral infection or an indirect immune-mediated mechanism (summarized in Figure 4) it appears that the behaviour of HAdV-F41 and AAV-2 is abnormal, in that species F adenoviruses do not infect liver cells and AAV-2 is not considered to be associated with a disease or produce clinical symptoms. It is possible, therefore, that mutations could have occurred to account for the changes in properties of the viruses. Several investigations have shown that mutation in the HAdV fibre gene can alter the tropism of adenoviruses [197–199, 200,201]. For example, the addition of a 7-lysine-residue motif at the C-terminal end of HAdV-F40 short fibre (which does not bind CAR) facilitated efficient transduction of target cells, which were not previously susceptible to infection, via the heparan-containing receptor [202]. In the studies of hepatitis of unknown origin, full or partial sequencing of the HAdV-F41 genome was carried out in many cases. For example, in one reportseveral early region proteins were found not to have significant mutations compared to control HAdV-F41 [144]. When the entire HAdV-F41 genome was sequenced, it was concluded either that SNPs were shared between cases and controls [144] or the patient HAdV-F41 sequence was similar to a previously reported virus [143]. Other partial HAdV-F41 sequences appear to vary slightly from previously published data but not to such an extent that it is likely to account for the virus’s change in tropism and/or its ability to cause novel disease. However, it is clear from the example of the omicron variant of SARS-Cov-2 that a single amino acid substitution can radically alter virus infectivity and severity of disease caused.

Possible mutations in AAV-2 could also change its ability to produce a deleterious infection. Sequencing of AAV-2 has also been carried out in detail. In one report, the genomes of AAV-2 were sequenced from 13 hepatitis patients [145]. Of 35 mutations, 43% were in the *AAP* gene, 40% in *VP1,* and 17% in *Rep78*. Perhaps, it is significant that over 70% of these mutations were the same as those identified in a second study [143]. In this latter investigation, it was noted that nine of the capsid gene mutations seen in the AAV-2 from hepatitis patients were associated with an AAV-2 variant (AAVv66) which has an altered phenotype [143,203]. AAVv66 has increased tissue spread, increased virion stability, and production of progeny, as well as an ability to evade neutralizing antibody [203]. In the third study identifying AAV-2 in the hepatitis patients, extensive sequencing of AAV-2 genomes showed little or no difference between cases and controls although it was noted that there were changes in the *capsid* gene in all contemporary samples which were absent from historic AAV-2s [144].

## Concluding remarks

Historically, interest in the enteric adenoviruses has stemmed from their ability to cause gastroenteritis and diarrhoea in children. This probably represents the most serious clinical condition due to any adenovirus species, in immunocompetent individuals. Specific studies of the properties of the viruses have been partially limited by difficulties in growing them in the laboratory because replication is restricted in several commonly used cell lines. Additionally, adenovirologists, when investigating viral replication, for example, or the biochemistry of particular adenovirus proteins, have tended to concentrate on those species for which most information is already available, such as HAdV-C5 and, to a lesser extent, HAdV-A12. Therefore, much of our knowledge of the life cycle and properties of HAdV-F41 has been based on the assumption that they are similar to those of HAdV-C5. This has been aided by the availability of nucleotide sequences for HAdV-F40 and HAdV-F41, allowing a comparison of theoretical protein primary structures with HAdV-C5 orthologs. Even so, the identity between HAdV-F40 and HAdV-C5 or HAdV-A12 for most early genes is less than 60%, suggesting possible marked differences in the mode of action [16].

The recent reports linking HAdV-F41 to severe, acute hepatitis in children has stimulated interest in the virus, to such an extent that cryo-EM structures are now available [17,18]. Some notable differences to HAdV-C5 have been observed, and it has been suggested that these may be responsible for the tropism of the enteric adenoviruses and their ability to withstand the low pH found in the gut. It would be beneficial if this renewed interest in HAdV-F41 could also be extended to a biochemical analysis of the properties of the early region proteins.

Regarding the causes of the severe hepatitis, there is no unequivocal explanation (Figure 4). The number of cases has decreased since the late summer of 2021 to pre-pandemic levels. However, it is notable that there have always been cases of childhood hepatitis, which could not be attributed to any of the common hepatitis viruses [139,140]. It is possible that they share an aetiology with the latest outbreak, although in one retrospective study there was little evidence of HAdV infection in the pre-2019 cases [189]. It seems reasonable to suppose that the COVID-19 pandemic may have contributed to the outbreak. Although most of the patients tested negative for SARS-CoV-2, at the time of hospitalization, it is possible that previous infections could have occurred. There is extensive evidence that SARS-CoV-2 can cause acute hepatitis (see, for example, [204] [205,206] [207]). In addition, it is clear that the prolonged lock-down and isolation have meant that very young children have reduced immunity. These factors, as well as a possible change in tropism of HAdV-F41 and perhaps alteration in responses to AAV infection, could, taken together, have led to an increase in acute hepatitis in children but without a single factor being responsible. Further investigation is required for a definitive explanation.

## Data Availability

All data mentioned and referenced in this review is available and open to use without restriction.
